# Ambient air quality and spatio-temporal patterns of cardiovascular emergency department visits

**DOI:** 10.1186/s12942-018-0138-8

**Published:** 2018-06-08

**Authors:** Eun-Hye Yoo, Patrick Brown, Youngseob Eum

**Affiliations:** 1 Department of Geography, University at Buffalo, Buffalo, NY USA; 2 Department of Statistical Sciences, University of Toronto, Toronto, Canada

**Keywords:** Cardiovascular disease (CVD), Emergency department (ED) visits, Hierarchical Bayesian model, Community multiscale air quality (CMAQ), Ambient air quality, Spatial effects

## Abstract

**Background:**

Air pollutants have been associated with various adverse health effects, including increased rates of hospital admissions and emergency room visits. Although numerous time-series studies and case-crossover studies have estimated associations between day-to-day variation in pollutant levels and mortality/morbidity records, studies on geographic variations in emergency department use and the spatial effects in their associations with air pollution exposure are rare.

**Methods:**

We focused on the elderly who visited emergency room for cardiovascular related disease (CVD) in 2011. Using spatially and temporally resolved multi-pollutant exposures, we investigated the effect of short-term exposures to ambient air pollution on emergency department utilization. We developed two statistical models with and without spatial random effects within a hierarchical Bayesian framework to capture the spatial heterogeneity and spatial autocorrelation remaining in emergency department utilization.

**Results:**

Although the cardiovascular effect of spatially homogeneous pollutants, such as PM2.5 and ozone, was unchanged, we found the cardiovascular effect of NO$_2$ was pronounced after accounting for the spatially correlated structure in emergency department utilization. We also identified areas with high ED utilization for CVD among the elderly and assessed the uncertainty associated with risk estimates.

**Conclusions:**

We assessed the short-term effect of multi-pollutants on cardiovascular risk of the elderly and demonstrated the use of community multiscale air quality model-derived spatially and temporally resolved multi-pollutant exposures to an epidemiological study. Our results indicate that NO$_2$ was significantly associated with the elevated ED utilization for CVD among the elderly.

## Background

Adverse health effects of air pollutants have been documented in numerous past studies, investigating the associations between various health outcomes and exposures to ambient air pollution [1–4]. Some studies focused on the impact of long-term accumulated exposures on chronic health outcomes, whereas others focused on the acute effect of exposure by exploring the associations between short term changes in air pollution exposure and daily deaths or hospital admissions. A number of studies of emergency department (ED) utilization, which is a relatively sensitive health outcome for respiratory conditions and cardiovascular illnesses [5], have also demonstrated the effect of increased ambient particulate matter on acute health outcomes [4, 6, 7].

It is worth noting, however, that the majority of studies that have investigated associations between air pollution exposure and emergency department visits were conducted using case-crossover or time-series designs [7–12], whereas little to no attention was given to geographical variations in ED use. In our review of 167 studies on health effects of air pollution published between 1999 and 2017, a total of 55 studies (33%) used a case-crossover approach, 106 studies (64%) used a variant of time-series design, and 4 studies utilized both study designs [13–16]. Our search was conducted in PubMed and ScienceDirect databases up to November 2017 using a combination of the following keywords: emergency department/room and air pollution. Among these studies, spatially and temporally varying air pollution exposure were considered only in 14 studies and these following studies by Carlin et al. [17], Zhu et al. [18], Wannemuehler et al. [19], and Sarnat et al. [20] assessed the effect of air pollution on geographical variations of ED utilization.

Perhaps the popularity of these two modeling approaches compared to spatial models might be due to the well-established associations between ambient air pollution and respiratory outcomes [21], but also the limited availability of spatially and temporally resolved air quality data. The population-weighted spatial average of measurements from monitoring sites have been used in both time-series analysis and case-crossover studies to approximate city-wide or regional average ambient concentrations. This approach is relevant as long as the spatial homogeneity assumption is met, but can lead to increased uncertainty and potential bias in their estimates of health risk when the spatiotemporal heterogeneity of pollutants is pronounced [20]. Meanwhile, considerable improvements have been made in spatially and temporally dynamic air quality modeling efforts, which include community multiscale air quality (CMAQ) model [22] and optimal aerosol depth values retrieved from remote sensing [23]. CMAQ is one of the most widely used regional air quality modeling systems, which has been used to evaluate pollution control measures and to determine source contributions to air pollutants, but also to provides air pollution exposure estimation in epidemiological studies [24, 25]. Recently Environmental Protection Agency (EPA) released fine scale predictions of pollutant levels, which were obtained by fusing monitoring data with the CMAQ model outputs. Although these spatially and temporally resolved pollutant surface estimates are subject to calibration bias and uncertainties [26–28], there is the potential of improving the quality of individual and population exposure to ambient pollution. Meanwhile, the applications of CMAQ related air quality data to population-level epidemiological studies are still rare with a few exceptions [29, 30].

The other issue in epidemiological studies on cardiovascular and respiratory effect of air pollution is that health associations with exposure to air pollutants are affected by neighborhood effects. A recent study by O’lenick et al. [31] reported that neighborhood-level socioeconomic status (SES) is a key factor that contributes to short-term vulnerabilities to air pollution-related respiratory morbidity, such as asthma, among children (5–18 years old). Likewise, Winquist et al. [32] argued that this SES effect is generalizable based on their multi-city study. However, it is still questionable if their neighborhood-level SES effects hold for different study locations, study periods, or health outcomes other than respiratory disease. Given that the vulnerability of subgroups to CVD is more pronounced than other types of diseases [33–35], it is necessary to account for the effect of neighborhood-level SES to identify the vulnerabilities among the most susceptible individuals to air pollution related CVD.

From a statistical standpoint, a Poisson process model appears to be a natural choice to explore geographic variation in ED use with respect to neighborhood health effects. However classical Poisson regression models may be problematic for ED visit counts aggregated over spatial units, such as zip codes. First, there might be missing or confounding variables that were not captured at the scale of analysis, which consequently may yield over-dispersion problems. Second, cardiovascular effects of air pollution are not likely to occur along the spatial boundaries of zip code units but rather smoothly change across boundaries of areal units. To reduce a bias in the model estimation and inference of cardiovascular effect of air pollution associated with a specific scale of analysis in the present study, we need a spatial model that explicitly address issues of spatially correlated structure in data.

In this paper, we aim to fill the gap in the literature by evaluating the short-term cardiovascular effect of ambient air pollution to the elderly, while increasing our understanding of the spatio-temporal patterns of CVD risks. We focused on the elderly in the present study because CVD related mortality is the most pronounced among this age group both in our preliminary data analysis and CVD related literature [4]. The associations between exposure to air pollution and CVD risks will be assessed after controlling for neighborhood characteristics that potentially impact patients’ health. To achieve our goal, we linked ED visit records for CVD of individuals age 65 years and above to spatially and temporally resolved multi-pollutant exposures derived from CMAQ models in Western New York, US, in 2011. We used a Bayesian hierarchical model to assess the effect of patients’ residential environments for physical activity and diet, as well as exposure to air pollutants on CVD risk, while accounting for spatial effects and uncertainty in the model inference.

## Methods

### Data and study area

The study area encompasses the Buffalo-Niagara region within Erie and Niagara counties of western New York, U.S. The records of Emergency Department (ED) visits for cardiovascular disease (CVD) were obtained from Statewide Planning and Research Cooperative System operated by New York State Department of Health. We focused on the records collected between January, 1, 2011, and December, 31, 2011. The original records contain information on admission date, discharge date, date of birth, 5 digit zip code of residence and demographic information (age, gender, ethnicity, and race) of individuals. We used residential 5 digit zip code and the day of visit as the finest spatial and temporal unit, respectively, for subsequent statistical analyses. The records also include the primary and secondary international classification of disease 9th (ICD-9) revision codes for diagnosis. Using the primary ICD-9 diagnosis code, we defined several cardiovascular disease groups. The diagnosis of CVD incorporates few sub-categories: hypertensive disease (401–405), ischemic heart disease (410–414), pulmonary heart disease (415–417), other forms of heart disease (420–429), cerebrovascular disease (430–438), and atherosclerosis (440). Our grouping and selection of records are largely based on published studies [3, 36].

Daily particulate matter with an aerodynamic diameter less than or equal to $2.5\, \upmu \hbox {g}/\hbox {m}^3$ (PM2.5) and ozone (O$_3$, in ppb) surfaces were obtained from the Downscaler (DS) model (https://www.epa.gov/air-research/downscaler-model-predicting-daily-air-pollution). The DS model fuses output from a gridded atmospheric model, the community multi-scale air quality model (CMAQ), with point air pollution measurements from fixed monitoring network, and predicts spatially and temporally resolved air quality, such as daily concentration at U.S. census tract centroid locations. To address the known issues of CMAQ estimates—CMAQ calibration bias and uncertainties [26–28], the DS model used a spatially-varying weighted model that regresses monitoring data on a derived regressor obtained by smoothing the entire CMAQ output with weights that vary both spatially and temporally. The DS model provides only PM2.5 and ozone, so we directly derived daily nitrogen dioxide (NO$_2$, in ppmv) levels from CMAQ model at $12 \times 12\,\hbox {km}$ resolution. Our research team conducted an extensive accuracy assessment of CMAQ model across western New York for 2011 [37]. To resolve the differences between the spatial unit of analysis—5 digit zip code units (Zips) and those at which pollutant data are available, we processed daily average of these multi-pollutant concentrations using GIS polygon overlay, more specifically, using the maximum function, to estimate daily air quality at Zips. Daily meteorological data were obtained from National Center for Environmental Information Climate Data Online system (http://www7.ncdc.noaa.gov/CDO/cdopoemain.cmd?datasetabbv=DS3505&countryabbv=&georegionabbv=&resolution=40) for two land-based monitoring stations within Erie/Niagara counties from January, 1, 2011 to December, 31, 2011. They include average temperature, dew point temperature, apparetus temperature, as well as relative humidity, wind speed and wind direction.

Socioeconomic data of the study region were obtained from 2010 Census and 2012 American Community Survey (ACS) at Zips. We summarized information on age- and gender-specific background population, and considered median household income, housing vacancies, education level less than high school graduate degree among adults over 18 years old, and health insurance coverage as proxy measures of poverty and economic status of each Zip. Health insurance coverage was quantified by calculating the percentage of population in each Zip who does not have health insurance coverage based on five year estimates between 2008 and 2012. Other SES variables were obtained from ACS five year estimates between 2007 and 2011. The one year gap between the health insurance coverage and other SES variables was due to the ACS data availability, as health coverage for the study area was published since 2012.

We also included the total number of healthcare facilities within Zips, which included hospitals, medical centers, federally qualified health centers, and home health services except nursing homes. The data from Department of Health and Human Services in 2012 were exploited to generate the locational information of healthcare facilities. The local food environment was characterized by the total number of grocery stores in each Zip. We obtained a list of businesses from 2016 InfoUSA (https://www.infousa.com) and identified grocery markets based on their business types, such as grocery-retail, grocery-wholesale, and food market. Using the information on their geographical coordinates, we counted the total number of grocery markets in each Zip. Similarly we assume that the spatial variation of local environment may promote a range of elderly populations’ physical activities [33, 38] and quantified the availability of areas designated as state, county, or municipal parks per Zip. Specifically, we quantified the availability of physical activity resources based on the following two GIS data sources—boundary of state parks and the tax parcels data of Erie/Niagara counties. Both of them were obtained from New York State GIS clearinghouse (https://gis.ny.gov). We extracted parcels of county/municipal park based on the property types of parcels, including public parks, playgrounds, picnic grounds, and recreational facilities. For each Zip, the total areas of the parks was calculated and divided by the area of the Zip, then multiplied by a hundred to derive the percentage of park areas.

### Ecological analysis: spatio-temporal models

Ecological time-series is a statistical approach established in environmental epidemiology to investigate the acute effect of air pollution [10]. Disease mapping has been used to elucidate the geographical distribution of underlying disease rates and to identify areas with low or high rates of incidences or mortality. However, the consideration of both spatial and temporal aspects of health outcomes with respect to the spatially and temporally varying air pollution is relatively rare. To fully explore the effect of air pollution while controlling spatially and temporally varying confounding factors, we developed a Bayesian hierarchical Poisson linear model for total counts of daily ED visits for CVD and used integrated nested Laplace approximations [INLA, see 39] for estimation.

Our goal was to assess potential cardiovascular effect of ambient air pollution exposure on the elderly and to identify areas with unusually high or low ED use. Total count of daily ED visits for CVDs among the elderly per Zip based on patients’ home address was used as a response and denoted as $Y_{it}$, $i=1, \ldots 84$, $t=1, \ldots , 365$. Given that the fraction of the population suffering from serious cardiac emergency on a given day is quite small, we assume that the count of independent events of ED visit that are randomly occurring in time follows the Poisson distribution [40]. Specifically, the observed daily ED visit counts $Y_{it}$ for CVD at a Zip was modeled using a Poisson likelihood as $Y_{it} \sim$ Poisson$(E_{i}\lambda _{it})$, where $E_{i}$ denotes the expected counts of daily ED utilization from unit *i* and $\lambda _{it}$ is the corresponding relative risk on day *t*. An expected count for Zips can be derived either from known national rates for CVD or from a more local standard population [41], but we obtained $E_{i}$ from age-specific standardized mortality ratio (SMR) using our ED records as $E_{i} = \sum _{j=1}^J n_{j,i} \hat{p}_j$ where $\hat{p}_j = \sum _i Y_{j,it}/\sum _i n_{j,i}$ denotes the observed overall visit rate for the age-group category $j = 1, \ldots , J$.

For the log-relative risk $\log (\lambda _{it})$, we identified potential risk factors of ED utilization for CVD via exploratory data analyses, which include a set of spatially and temporally resolved air pollution exposure variables $X_{k}, k=1, \ldots , 3$. Our exploratory analysis indicated that there are considerable amounts of multicollinearity among weather conditions with a linear trend/a within-year cyclical pattern and among SES variables including housing vacancy/education achievements and median household income, and we omitted variables with weak correlation with ED visits. The final model consisted of temporal variables ${T}_{l}, l=1, \ldots , 12$ to capture temporal pattern of ED utilization among the elderly, and spatial variables $Z_{m}, m=1, \ldots , 5$ to represent socioeconomic conditions of patients’ residential location and their local environments for physical activity and diet, and is written as

1 $$\begin{aligned} \log (\lambda _{it}) = \beta _0 + \sum _{k=1}^3 \beta _k X_{k,it} + \sum _{l=1}^{12} \alpha _l {T}_{l,t}+ \sum _{m=1}^5 \eta _m Z_{m,i} + U_i \end{aligned}$$

Here, $\beta _0$ is an intercept term, and $\beta _1, \beta _2, \beta _3$ denotes the effect of daily NO$_2$, ozone, and PM2.5 exposure in Zip *i* during day *t*. Regarding the daily pattern of ED utilization among the elderly, we found that a within-year cyclical pattern is present, visits vary by day of the week, and there appears to be an overall declining trend. Specifically, the explanatory variables included in ${T}_{l}$ are following:

- a within-year cyclical pattern, represented as two sine and two cosine functions with periods of 12 and 6 months evaulated at time *t*;
- a day of the week effect with 8 levels (7 days plus one level for holidays) at time *t*;
- a linear trend;

Following Diez-Roux [34], we hypothesized that geographic variations of ED utilization are associated with socioeconomic status, the accessibility to healthcare facilities, and local environments for physical activities and diet. These Zip-specific covariates $Z_m$ include

- Median household income and health insurance coverage at the Zip *i*
- Total number of healthcare facility, except nursing homes
- Percentage of park area
- Total number of grocery markets

The spatial random effects, denoted as $U_i$, are fully structured to accommodate spatial dependence through a Besag-York-Mollie [or BYM, see 42 model as $(U_1 \ldots U_N)' \sim$ BYM$(\sigma _1^2, \sigma _2^2)$, where the Zip-specific random effects $U_i$ are designed to capture extra-Poisson variability in the observed ED visit rates. These random effects $U_i$ are modeled as a sum of a spatially structured component $V_i$ (or more specifically, a first-order Markov random field) and a spatially independent term $W_i$. The independent term is modeled as $W_i \sim N(0, \sigma _2^2)$ thus modeling overall heterogeneity [18], while the spatial term is defined using a conditionally autoregressive (CAR) specification as

2 $$\begin{aligned}{}[ V_i| V_j, j \ne i] \sim N\left( \frac{\sum _{j \sim i} V_j }{| j \sim i|}, \frac{c \sigma _1^2}{ |j \sim i| } \right) \end{aligned}$$

where $j \sim i$ indicates that *j*-th Zip is a neighbour of region *i* with at least one boundary point in common. Following Simpson et al. [43], *c* is a scaling factor making $\sigma _1^2$ approximately equal to the marginal variance of $V_i$ and penalized complexity prior distributions are assigned to combinations of the two variance parameters. The sum of the variances (square-rooted) has an exponential prior distribution with $pr\left( \sqrt{\sigma _1^2 + \sigma _2^2} > 0.5 \right) = 0.1$, a fairly uninformative prior which on the upper end of 0.5 will give log relative risks as large as 1.0 and as small as − 1.0 with corresponding relative risks close to 3 and 1 / 3. The fraction of the variation due to the spatial process has a penalized compliexity prior with $pr\left( \sigma _1 / \sqrt{\sigma _1^2 + \sigma _2^2} < 0.1\right) = 0.8$, favouring a spatially independent model (where this fraction is zero) but allowing for a large degree of spatial dependence (with the fraction close to one) should the data warrant it. The BYM model was fitted using the diseasemapping package (v 1.4.2) in R (v 3.4.0).

As a diagnostic tool, a zero-inflated Poisson distribution was substituted for the Poisson distribution for incidence counts. A large portion of the daily visit counts are zero, and the zero-inflated model introduces an additional parameter to induce more zeros than the Poisson distribution allows for. Were the Poisson model correct, and zeros are meerly the result of small expected counts for daily data at the Zip level, the estimate of the zero-inflation parameter would be expected to be small. This model was implemented as the zeroinflated1 distribution in INLA, with the zero-inflation parameter having a Beta(1,9) distribution.

## Results

### Descriptive statistics of data

The characteristics of ED utilization in the study area during 2011 are summarized in Table 1. The gender differences in the ED visit for CVD are not substantial unlike the differences by age group. Individuals age over 65 take the majority of the 2011 ED utilization (46.0 %) compared to their demographic composition (15.8%) in this region. The seasonal differences of ED utilization are not substantial. Although the proportion of ED utilization in summer and winter (33.0 and 33.2%) are about twice large as those of spring and fall (16.7 and 17.0%), the summer and winter months are defined as four months, whereas the spring and fall are defined as two months based on the seasonal variability in the study region. The effect of the day of week is more noticeable such that Tuesday and Wednesday are the lowest (12.3 and 11.6%) and Sunday and Monday are highest (16.2 and 15.2 %).

**Table 1:** ED utilization by age group, sex, season, and day of week

	Patients (%)	Population (%)
All ages	5798	1,135,474
*Sex*		
Female	2997 (51.7%)	587,469 (51.7%)
Male	2801 (48.3%)	548,005 (48.3%)
*Age*		
0–64	3136 (54.1%)	955,556 (84.2%)
65–74	983 (17.0%)	87,569 (7.7%)
75+	1679 (29.0%)	92,349 (8.1%)
*Aged over 65*	2662	179,918
*Sex*		
Female	1549 (58.2%)	106,292 (59.1%)
Male	1113 (41.8%)	73,626 (40.9%)
*Season*		
Winter	879 (33.0%)	
Spring	445 (16.7%)	
Summer	885 (33.2%)	
Fall	453 (17.0%)	
*Day of week*		
Sunday	431 (16.2%)	
Monday	404 (15.2%)	
Tuesday	339 (12.7%)	
Wednesday	313 (11.8%)	
Thursday	393 (14.8%)	
Friday	409 (15.4%)	
Saturday	373 (14.0%)	

**Fig. 1:**
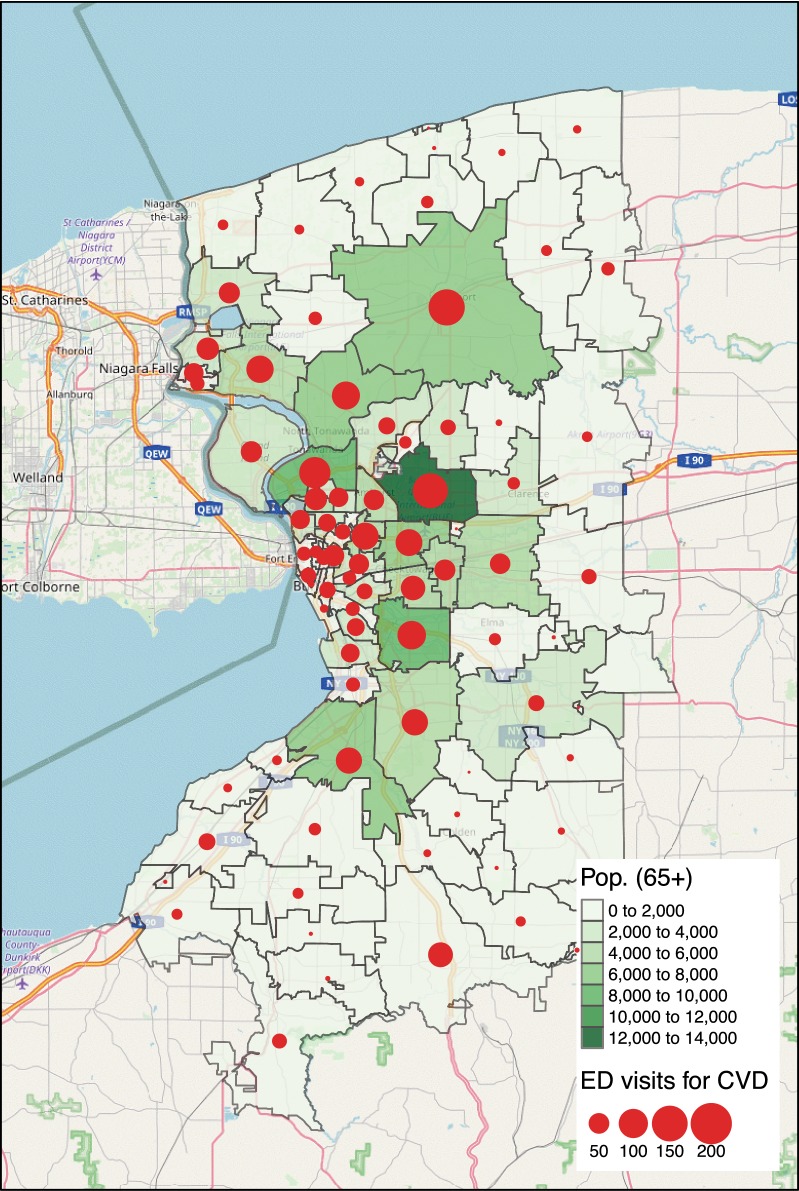
Spatial distribution of population age over 65 and ED visits for CVD

The spatial distribution of the ED utilization in 2011 was examined under the consideration of the underlying population at risk, the spatial distribution of the elderly age over 65 at each Zip. The size of the elderly per Zip varies from 17 to 12,680 people with the mean of 2192 and the standard deviation 2370. The spatial distribution of the elderly in Fig. 1 shows that the elderly resides in suburbs around the city of Buffalo forming a ring pattern centered at the city center. The Zips with more than 2829 elderly residents (75th percentile) have the median household income between $31,383 and $80,302, which correspond to 0.25 quantile and 0.75 quantile of the regional median household income levels. The raw counts of daily ED visit per Zip vary from zero to three with an average 0.09 with a standard deviation of 0.31 (see Table 2). The spatial distribution of ED utilization was quantified by aggregating counts of ED visits per Zip over the year 2011 and visualized in bubble plot of Fig. 1. The size of bubbles is proportional to the total counts of cadiovascular ED visit in 2011. As expected, this pattern is strongly correlated (*r* = 0.92) with the spatial distribution of the elderly. A few exceptions were found in the south and south west areas of the Erie county, where relatively high ED visits were observed despite their small population. The temporal distribution of ED utilization is characterized by a mean of 7.29 cases per day with the standard deviation 2.75. The day with the highest utilization in 2011 had a total of 16 cases across the study region.

**Fig. 2:**
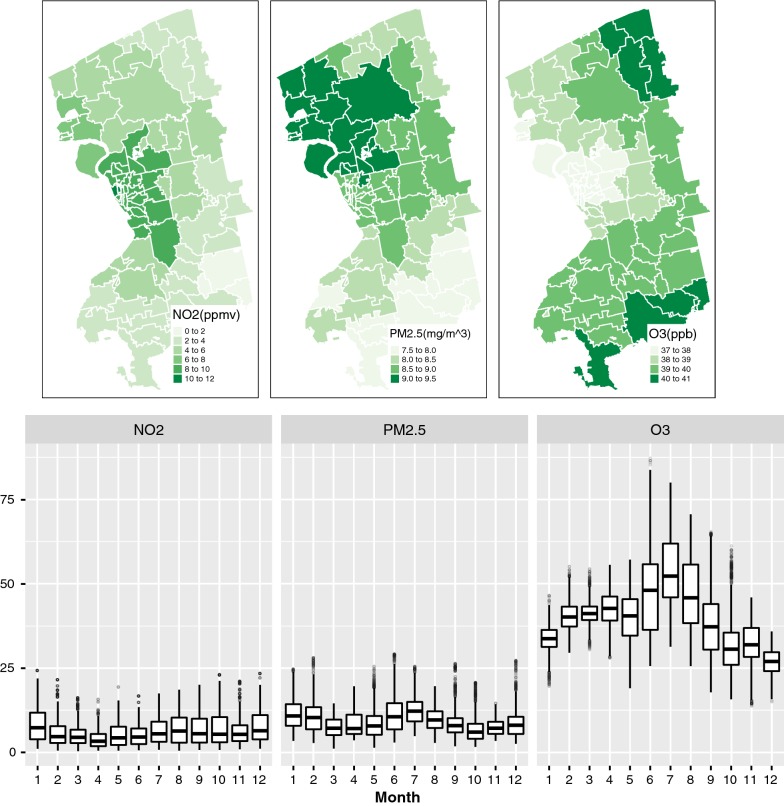
Spatial and temporal variability of ambient pollutant concentrations

The distribution of the three air pollutants are summarized in Table 2 and mapped in Fig. 2. Zip level daily NO$_2$ ranged from 0.38 to 24.29 ppmv with a mean of 6.26 ppmv and the standard deviation of 4.31. Similarly, daily PM2.5 concentrations at Zip ranged from 1.13 to $29.22\,\upmu \hbox {g}/\hbox {m}^3$ with a mean of $9.48 \,\upmu \hbox {g}/\hbox {m}^3$ and standard deviation of 4.76. Both PM2.5 and NO$_2$ have relatively small variability year-round as shown in monthly time scale of box plots (see Fig. 2), although their spatial patterns are quite different from each other. High concentrations of NO$_2$ are centered at the city of Buffalo where high traffic volume exists, while PM2.5 is high at the north west of the study area including Tonawanda in which a violation of the Clear Air Act by Tonawanda Coke Corporation was reported [44, 45]. Daily ozone concentration shows a cyclical pattern—high in summer months with a peak in July and low in cold months with the mean and standard deviation 39.63 ppm and 10.81, respectively, and a wider range of lowest value 13.77 and maximum 87.21 ppb. This seasonal variability is observed in both PM2.5 and ozone, but is slightly different as ozone is lower in both spring and winter whereas the PM2.5 is lowest in spring and fall. The spatial pattern of ozone is quite different from PM2.5 and NO$_2$, as the high concentration of ozone is found in east side of the study area and lowest at the city of Buffalo (see Fig. 2).

**Table 2:** Spatio-temporal distribution of cardiovascular ED visits and air pollutants

	Mean ± SD	Min.	1st Q.	Median	3rd Q.	Max.
*ED visits*						
Daily counts per zip	0.09 ± 0.31	0.00	0.00	0.00	0.00	3.00
Total counts per zip	31.69 ± 32.39	0.00	7.75	22.50	45.75	153.00
Total counts per day	7.29 ± 2.75	0.00	5.00	7.00	9.00	16.00
*Air pollutants*						
NO$_2$ (ppmv)	6.26 ± 4.31	0.38	2.87	5.15	8.59	24.29
PM2.5 ($\upmu \hbox {g}/\hbox {m}^3$)	9.48 ± 4.76	1.13	5.88	8.60	12.00	29.22
Ozone (ppb)	39.63 ± 10.81	13.77	31.87	38.65	45.44	87.21

### Spatio-temporal ecological models

To allow a proper assessment of the spatio-temporal ecological model, we began fitting a Poisson generalized linear model (GLM) with the full set of covariates used for the spatio-temporal random effect model in Eq. (1). The model fits are summarized in Table 3.

**Table 3:** Fitted relative risk for the parameters of interests via Poisson regression and spatial random effect model

	Poisson regression model				Spatial random effect model			
	Mean	0.025Q	0.975Q	SD	Mean	0.025Q	0.975Q	SD
(Intercept)	*1*.*16*	1.04	1.29	1.06	*1*.*19*	1.06	1.35	1.06
sin12	*0*.*89*	0.80	0.99	1.05	0.92	0.83	1.02	1.06
cos12	1.02	0.95	1.09	1.04	1.00	0.93	1.07	1.04
sin6	0.99	0.92	1.07	1.04	0.99	0.92	1.06	1.04
cos6	0.98	0.92	1.03	1.03	0.97	0.91	1.02	1.03
Tuesday	1.00	0.86	1.15	1.07	0.99	0.86	1.14	1.07
Wednesday	0.90	0.78	1.04	1.08	0.91	0.79	1.05	1.08
Thursday	0.97	0.84	1.12	1.08	0.97	0.84	1.12	1.08
Friday	1.05	0.91	1.21	1.07	1.06	0.92	1.21	1.07
Saturday	*0*.*80*	0.69	0.93	1.08	*0*.*82*	0.71	0.96	1.08
Sunday	*0*.*76*	0.65	0.88	1.08	*0*.*79*	0.68	0.92	1.08
Holidays	*0*.*68*	0.49	0.93	1.18	*0*.*71*	0.51	0.98	1.18
Day	0.77	0.58	1.01	1.15	0.78	0.59	1.04	1.15
Median Income	*0*.*88*	0.81	0.95	1.04	*0*.*80*	0.70	0.92	1.07
% No-insurance	*1*.*13*	1.03	1.24	1.05	1.00	0.86	1.16	1.08
No. Health Fac.	*1*.*04*	1.01	1.07	1.01	1.05	0.98	1.12	1.03
% Green space	0.99	0.94	1.04	1.03	1.01	0.92	1.12	1.05
Grocery stores	*0*.*94*	0.90	0.99	1.02	0.97	0.88	1.06	1.05
NO$_2$	0.93	0.83	1.04	1.06	*1*.*15*	1.01	1.30	1.06
PM2.5	1.00	0.95	1.05	1.03	1.00	0.95	1.06	1.03
O$_3$	0.97	0.87	1.07	1.05	0.92	0.83	1.02	1.06
Non-spatial					1.40	1.29	1.55	
$$\sqrt{\sigma _1^2 + \sigma _2^2}$$								
Spatial					2.14	1.40	2.66	1.19
$$\sigma _1 / \sqrt{\sigma _1^2 + \sigma _2^2}$$								
DIC	16427.89				16264.55			

The significance of italics was determined based on the 95% credible intervals for the fixed effects (known risk factors)

**Fig. 3:**
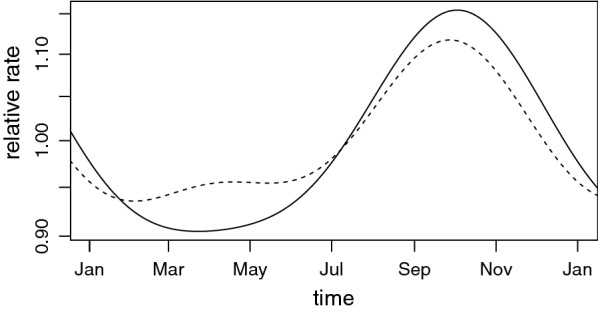
Estimated seasonal effect for Poisson regression (straight line) and spatial (dashed line) models

Compared to the ED utilization on Mondays, a low utilization of emergency department visits for CVD was observed on both weekend and holidays with statistical significance. A cyclical term for sine 12 was also significant. Figure 3 shows the estimated seasonal effect for both the Poisson GLM and the spatial random effects model. The estimated coefficients for the sine and cosine functions in Table 3 determine these seasonal effect. Both models agree that the period of peak ED use is from September to November, with February to June being the time of year with the fewest ED visits.

Among the spatial covariates associated with socioeconomic status, both the median household income and the total number of healthcare facilities located in each Zip have statistically significant associations with ED utilization. Strong and negative association with median household income corroborates the previous finding that high ED utilization pattern is an indicator of poor economic status at community level [46], meanwhile a small but positive association with healthcare facilities might be associated with the fact that our study is purely based on the elderly who prefer to reside near healthcare facilities. In terms of neighborhood environments for physical activities, we did not find the percentage of green space in each Zip being significant at 95% credible intervals. The number of grocery stores in each Zip was negatively associated with the high ED use as one would expect. In the Poisson GLM, none of the three air pollutants were significant. For a purpose of model validation, we examined the residuals of the Poisson GLM for the possible presence of autocorrelation in space and time. First we computed the temporal autocorrelation function of daily residuals aggregated over the entire study area and found no significant temporal auto-correlation remaining in the Poisson GLM. On the other hand, Moran’s I index [47], a summary statistic widely used to evaluate the presence of spatial autocorrelation, was 0.381 with the p-value of 0.0001. This result suggested that there is considerable spatial autocorrelation in the residuals and an inclusion of a spatial correlation structure in the model will be appropriate.

For the spatial random effect model in Eq. (1), the model coefficient estimates are similar to the Poisson GLM for the temporal covariates but not for neighborhood health effects, including daily exposure to air pollutants. The positive associations observed in Poisson GLM between the neighborhood level accessibility to healthcare facilities/the percentage of residents without health insurance coverage and high ED use are no longer significant, but the positive effect of NO$_2$ exposure became significant after accounting for the spatial correlation in ED utilization at Zips. An increase of 10 unit of NO$_2$ exposure is associated with an increase of 15.00 % in the relative risk of ED utilizations for CVD among the elderly according to the Bayesian hierarchical model.

The estimate of the spatially-structured contribution to the Zip-level variation in ED use, shown as $\hat{\sigma }_1/ \sqrt{\hat{\sigma }_1^2 + \hat{\sigma }_2^2}$ in Table 3, suggests that non-trivial spatial patterns exist in ED utilizations. The moderately large estimate of 0.76 and the very large upper 97.5% quantile of 0.98 indicate the spatial model should be trusted over the Poisson GLM. The latter ignores all this spatially structured random error, which is likely to result in incorrect (narrower) estimates of uncertainty intervals of parameters that we are interested in, such as NO$_2$.

The zero-inflated model produced estimates nearly identical to those of the Poisson model (results appear in the Appendix), with the zero-inflation parameter having a posterior mean of 0.04. This suggests the Poisson assumption is appropriate and there are no ’structural’ effect inducing excess zeros.

### Mapping relative risk of cardiovascular disease

**Fig. 4:**
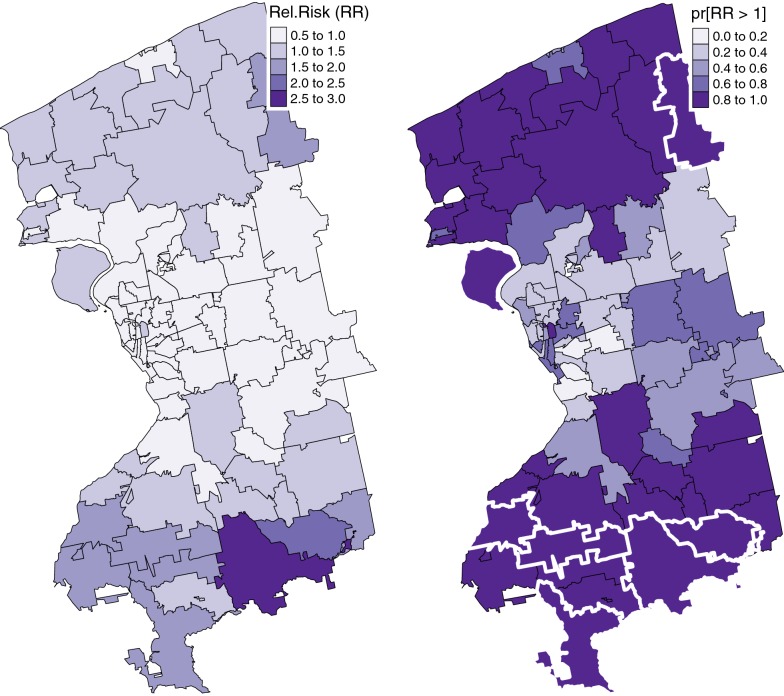
Posterior mean $\hbox {E}[\exp (U) | Y]$ and risk-exceedance probabilities $pr[\exp (U) > 1| Y]$ of the spatial random effects. The white thick line denotes the Zips with significant relative risk according to 95% credible intervals

In a Bayesian hierarchical model the relative risk of a disease is estimated as a posterior distribution instead of a single value. However, Bayesian disease mapping analysis results are typically presented as a map of a point estimate (usually the mean or median of the posterior distribution) of the relative risk for each area. To interpret such maps, one needs to understand the extent to which the statistical model is able to smooth the risk estimates to eliminate random noise while at the same time avoiding over-smoothing that might flatten any true variations in risk [48]. Figure 4a presents the posterior mean for the Zip-level random effect of relative risk estimates of cardiovascular disease of elderly in Erie/Niagara counties. The total number of Zips with relative risk above the overall average (Relative Risk > 1) was a total of 37, which corresponds to 44.04 %. The majority areas in the north and south of the study area have the high relative risks including one Zip in the south whose risk is above more than double of the region-wide overall risk. It is also noticeable that areas around the city of Buffalo have a relatively lower risk of CVD despite the considerable number of elderly residents as shown in the background map of Fig. 1.

To assess the uncertainty associated with the point estimate (posterior mean) of relative risk, we followed Richardson et al. [48] and Blangiardo and Cameletti [49] and mapped the likelihood of excessive risk based on the posterior probability (termed as “risk-exceedence probabilities”). That is, the probability that the relative risks (or spatial random effects) are greater than the region-wide risk, i.e., $p\{\exp(U_i) > 1|(y_1, \ldots , y_N)\}$, is visualized in Fig. 4b. The risk-exceedence probabilities associated with the 37 areas whose Relative Risk $> 1$ ranged between 0.82 and 1.00 with mean and standard deviation of 0.97 and 0.05, respectively. These high risk-exceedence probabilities imply that the uncertainty associated with the relative risk estimates of these Zips are quite small. We also identified Zips with significant relative risks based on the 95 % credible intervals of relative risk estimates. The results are shown in Fig. 4b using extra thick border lines. The results indicated that a total of 9 Zips with statistical significance relative risk all also had high risk-exceedence probabilities.

## Discussion

We explored the spatio-temporal variability of ED utilizations for CVD in relation to the spatial variation of daily exposure to air pollutants, such as NO$_2$, PM2.5 and ozone, at Zips. Our study advanced previous findings in western New York [50–52] in that we assessed the association between air pollution and ED utilization using spatially and temporally resolved air pollution exposure derived from the state-of-art atmospheric models (CMAQ). In a closely related study Castner et al. [52] assessed the short-term health effect of daily concentrations of multi-pollutants, including Carbon monoxide (CO), NO$_2$, PM2.5, and ozone, using a region-wide ambient air quality based on measurements obtained from a small number of monitors (two to five depending on the pollutant types). We argue that such approach may be appropriate for pollutants with limited spatial and temporal heterogeneity, but is problematic for certain pollutants, such as NO$_2$ or CO, which exhibit significant spatial heterogeneity [53]. Castner et al. [52] found no significant associations between ED asthma utilization and air pollution, but a positive and significant effect of NO$_2$ on ED utilization among the elderly for CVD was found in the present study. This difference might be due to that we have examined only one year (2011) instead of multi-year data (2007–2012), or the focus on different health outcomes, specifically, CVD rather than asthma. However, it is also possible that the explicit consideration of geographic variations of ED utilization and the spatially heterogeneity of air pollutant concentrations played a crucial role in revealing the CVD effects of NO$_2$ among the elderly.

To properly assess the cardiovascular effect of pollutants, we fitted two models with and without spatial random effects. Both models indicated that community level socioeconomic status is a determinant of ED utilization for CVD. The Poisson GLM also suggested that the associations with known spatial risk factors, such as the accessibility to healthcare facilities and the reduced access to healthy food options in Zips, were significant in addition to the temporal trend. Our findings on these significant temporal covariates concur with the existing literature [54–56] including the recent work by Castner et al. [51] that the day of week is the most influential predictor of ED utilization. As argued by Wargon et al. [56], the Monday effect appears to be a common driver that increases adult ED utilization across different study areas and study periods. This effect might be attributed to the return of patients from a weekend absence or return of primary care practitioners to their office and sending their patients to ED.

**Fig. 5:**
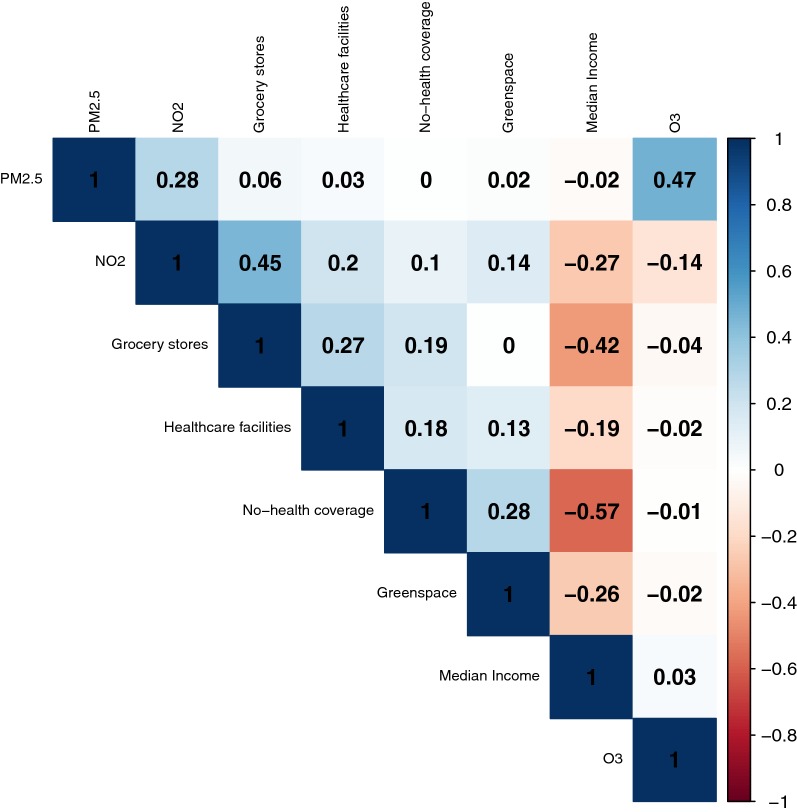
Pearson correlation matrix of risk factors

We found the spatial random effect model was more effective to investigate the spatial pattern of ED utilizations of the elderly in the study area than a Poisson GLM from the following reasons. Both the Moran’s I index of Poisson GLM and the conditional autoregressive specification parameter estimate from the BYM model suggested that there was a statistically significant autocorrelation in the residuals of Poisson GLM. We suspect that the presence of spatial autocorrelation is a natural outcome of using aggregated data—both for ED utilization and neighborhood-level covariates. However, the presence of spatial heterogeneity and autocorrelation might be associated with the measures of residential environments for physical activity, diet, and air quality in our study. We used Zip as a unit of analysis and summarized other covariates, including exposure to air quality and residential environments for green space and healthy food access, within the unit, but other spatial scales might have been more relevant to properly capture geographic variations of covariates. The literature on spatial statistics [41, Chap. 4 and 5] warns that the spatial scale or unit of analysis may induce the spatial heterogeneity and/or autocorrelation, as unmeasured covariates do. After accounting for the spatially correlated structure, we found that the neighborhood level exposure to NO$_2$ was positively associated with the risk of CVD, but several spatial risk factors, such as the percentage of people without health insurance, the number of healthcare facilities, and the number of grocery stores, were no longer significant in the spatial random effect model. One may suspect these changes are due to correlations between NO$_2$ concentration and spatial risk factors or the population at risk considered in our study. However, Fig. 5 indicated that the correlation between NO$_2$ and spatial risk factors, including the number of healthcare facilities at Zips, was low in the range of − 0.27 and 0.14 except the accessibility to grocery stores (0.45). Meanwhile, the strong negative correlation between median income and risk factors (up to − 0.57 with no health insurance coverage) and the noticeable changes in the effect median income from − 0.13 in Poisson GLM to − 0.22 in the spatial random effect model might affect the results. In addition, the target population considered in this study was elderly whose health insurance is covered by Medicare.

Mapping the area-specific random effects for CVD of the elderly suggests that the CVD risk is spatially heterogeneous even after we accounted for the geographic variation of the spatial and temporal risk factors. Interestingly the areas with high CVD risk above the region-wide risk (Relative Risk $> 1$) are concentrated in north and south of the study region with one Zip of the highest risk 2.92 (almost three times higher than the overall ED utilization for CVD) in the south border. This result is rather surprising because the raw ED visit counts in Fig. 1 show high ED utilization around the first ring around the city of Buffalo and small ED visits at the outer edges where the elderly is sparse. After taking into account demographic characteristics and risk factors together, the north and south side of the study regions were identified as having high ED utilization for CVD among the elderly. To fully understand the outputs from the Bayesian hierarchical model, we also visualized the uncertainty associated with relative risk estimates as shown in Fig. 4b. Based on this uncertainty assessment, we concluded that our identification of Zips with high cariodvascular risk are almost certain. Our study is not free of limitations and a number of issues still remain unresolved. First, potential errors in CMAQ-based air pollution exposure estimates have not been explicitly discussed in our study. Our decision is based on the fact that many studies including those sponsored by EPA (https://www.epa.gov/hesc/rsig-related-downloadable-data-files) and the Centers for Disease Control and Prevention (https://ephtracking.cdc.gov/DataExplorer/#/) have used spatially fused air quality surface in their regulation and decision making processes, but also our team’s recent study was directly related with the sensitivity assessment of CMAQ outputs and calibration [37]. We are confident that the quality of our CMAQ modeling outputs is sufficient to be used in epidemiological studies. Second, the present study related residential environments to observed cardiovascular risk, in which individual-level characteristics, such as existing chronic conditions, smoking and leisure time physical activities, were not statistically controlled. This problem, referred to as residual confounding, is common to ecological studies, and requires caution in interpreting results [34]. Third, we found that residential environments for diet and physical activities were not significant despite the growing literature supporting their associations. We suspect that the lack of associations might be related with measurements of such environments and the spatial scale at which those characteristics were captured. For example, aesthetically pleasing environment might be more encouraging physical activity participation for the elderly rather than a mere measure of accessibility to or availability of green space [57]. Similarly, the spatial scale of analysis at which both health outcome and spatial risk factors are characterized might have played an important role to determine their associations. The other critical issues lie in the potential multicollinearity among covariates. For example, Zip level ozone and PM2.5 are strongly correlated as median household income is negatively associated with health insurance coverage and the percentage of green space. These correlated variables may induce bias in the estimated model coefficients. The correlation among multi-pollutants is of particular concern. Our exploratory analysis indicates that the strong positive correlation (0.47) exists between O$_3$ and PM2.5 (see Fig. 5), but the correlations between NO$_2$ and the other two pollutants are relatively weak as − 0.14 for O$_3$ and 0.28 for PM2.5, respectively. This suggests that our finding on the effect of NO$_2$ on the ED utilization for CVD is less likely to be affected by the collinearity problem. We also evaluated the collinearity problem by running three single-pollutant models and compared these results with those from the proposed multi-pollutant models. We found that only NO$_2$ was significant at 90 % credible intervals, and none of the individual pollutants was significant at 95% credible intervals. We concluded that the collinearity was present among the three pollutants, but it was not influential in the present study. Liverani et al. [58] have developed profile regression specifically to address this problem of severe correlation among covariates, and we are taking this approach in future work to investigate how multicollinearity among covariates affects or modifies the cardiovascular effect of air pollution. We will be able to better understand the role of neighborhood health effects using profile regression. Lastly we will extend our study to multi-year ED utilization data in future work along with corresponding CMAQ driven multi-pollutant profile to evaluate our hypothesis on the cyclical patterns of ED visits.

Our findings have multiple public health implications. We identified neighborhoods with unusually high ED utilization for CVD among the elderly in the present study. The investigation of the factors responsible for health disparities in the study region and taking measures to improve health and healthcare delivery, increase access to care should be a topic for further investigation. One of our long-term goals is to translate our findings to local practice, that is, to improve population health. Our study parallels with on-going community efforts, such as Keys to Health (http://www.pophealthwny.org/), to develop preventive strategies, such as health education or chronic care managements, for residents in Western New York whose socioeconomic status and quality of health vary geographically. Our findings can be further used to effectively reallocate local healthcare resources to address population at greater risk, such as the elderly and the individuals with chronic diseases. We expect that dissemination of information on alternative healthcare facilities other than ED and the improvement on the accessibility of the elderly to non-ED health facilities may alleviate the ED burden. Lastly, our result suggests that NO$_2$ has significant associations with cardiovascular ED utilization among the elderly in the study region, and more stringent control for emission sources for NO$_2$ below normal health guidelines is needed to protect them.

## Conclusions

We assessed the short-term effect of exposure to NO$_2$ on the risk of ED visits for cadiovascular related disease among the elderly using a Bayesian hierarchical model. The model fit indicated there is a statistically significant association between daily exposure to NO$_2$ and the elevated ED utilizations for CVD when the spatial autocorrelation in the observed ED visit counts is accounted for. The results also indicated that there are areas with unusually high ED utilization for CVD compared to region-wide overall use of ED with little uncertainty. While our findings have the potential to be useful to improve our understanding of both the CVD risk among the elderly in the western New York and spatial disparity in ED utilization for CVD, further investigation is warranted to explore the multidimensional aspect of associations between air pollution exposure and ED visits.

## Appendix

The ED utilization data have more zeros than expected, based on the Poisson distribution. To account for the extra zero in daily ED uses we developed a zero-inflated Poisson regression model and the results are summarized in Table 4. The model fit is nearly identical to the spatio-temporal model in Table 3, which indicated that the spatio-temporal Poisson model is appropriate for the data.

**Table 4:** Fitted relative risk for the parameters via zero-inflated Poisson regression model

	Mean	0.025Q	0.975Q	SD
(Intercept)	*1*.*25*	1.10	1.44	1.07
sin12	0.92	0.83	1.02	1.06
cos12	1.00	0.93	1.07	1.04
sin6	0.99	0.92	1.06	1.04
cos6	0.97	0.91	1.02	1.03
Tuesday	0.99	0.86	1.14	1.07
Wednesday	0.91	0.79	1.05	1.08
Thursday	0.97	0.84	1.12	1.08
Friday	1.05	0.92	1.21	1.07
Saturday	*0*.*82*	0.71	0.96	1.08
Sunday	*0*.*79*	0.68	0.92	1.08
Holidays	*0*.*71*	0.51	0.98	1.18
Day	0.78	0.59	1.03	1.15
Median income	*0*.*80*	0.70	0.92	1.07
% No-insurance	1.00	0.87	1.16	1.08
No.Health Fac.	1.05	0.99	1.12	1.03
% Green space	1.01	0.92	1.12	1.05
Grocery stores	0.97	0.88	1.06	1.05
NO$_2$	*1*.*14*	1.01	1.30	1.06
PM2.5	1.00	0.95	1.06	1.03
O$_3$	0.92	0.83	1.02	1.06
Non-spatial	1.40	1.29	1.56	
Spatial	2.15	1.42	2.66	1.19
DIC	16266.54			

The significance of italics was determined based on the 95% credible intervals for the fixed effects (known risk factors)
